# The C-terminal region affects the activity of photoactivated adenylyl cyclase from *Oscillatoria acuminata*

**DOI:** 10.1038/s41598-019-56721-3

**Published:** 2019-12-30

**Authors:** Minako Hirano, Masumi Takebe, Tomoya Ishido, Toru Ide, Shigeru Matsunaga

**Affiliations:** 1 Bio Photonics Laboratory, The Graduate School for the Creation of New Photonics Industries, 1955-1 Kurematsu Nishi-ku, Hamamatsu, Shizuoka 431-1202 Japan; 2 Central Research Laboratory, Hamamatsu Photonics K.K., 5000 Hirakuchi Hamakita-ku, Hamamatsu, Shizuoka 434-8601 Japan; 3 Graduate School of Interdisciplinary Science and Engineering in Health Systems, Okayama University, 3-1-1 Tsushima-naka, Kita-ku, Okayama-shi, Okayama 700-8530 Japan

**Keywords:** Biophysical chemistry, Biophysics

## Abstract

Photoactivated adenylyl cyclase (PAC) is a unique protein that, upon blue light exposure, catalyzes cAMP production. The crystal structures of two PACs, from *Oscillatoria acuminata* (OaPAC) and *Beggiatoa* sp. (bPAC), have been solved, and they show a high degree of similarity. However, the photoactivity of OaPAC is much lower than that of bPAC, and the regulatory mechanism of PAC photoactivity, which induces the difference in activity between OaPAC and bPAC, has not yet been clarified. Here, we investigated the role of the C-terminal region in OaPAC, the length of which is the only notable difference from bPAC. We found that the photoactivity of OaPAC was inversely proportional to the C-terminal length. However, the deletion of more than nine amino acids did not further increase the activity, indicating that the nine amino acids at the C-terminal critically affect the photoactivity. Besides, absorption spectral features of light-sensing domains (BLUF domains) of the C-terminal deletion mutants showed similar light-dependent spectral shifts as in WT, indicating that the C-terminal region influences the activity without interacting with the BLUF domain. The study characterizes new PAC mutants with modified photoactivities, which could be useful as optogenetics tools.

## Introduction

Photoactivated adenylyl cyclase (PAC), which produces cAMP by blue light illumination, is an attractive tool to non-invasively regulate cellular events with precise spatial and temporal control. To date, several types of PACs have been identified from both prokaryotes and eukaryotes and have been used as optogenetic tools to control intracellular cAMP. PAC from *Euglena gracilis* (euPAC), which was the first to be discovered, is composed of two subunits, PACα and PACβ, forming a hetero-tetramer^1^. Each subunit consists of two light-sensing domains (BLUF domains) with a flavin molecule and two adenylyl cyclase (AC) domains. In previous studies, euPAC has been utilized to regulate cAMP levels in *Aplysia* sensory neurons, adult fruit flies, dentate granule cells, etc.^2–4^. Recently, PACs smaller than euPAC have been found, such as those from *Oscillatoria acuminata* (OaPAC) and *Beggiatoa* sp. (bPAC)^5–10^, which have expanded the use of PAC as an optogenetic tool and in the control of intracellular cAMP levels by blue light in hippocampal neurons, *Caenorhabditis elegans*, pancreatic β-cells, etc.^7,10–14^.

**Figure 1:**
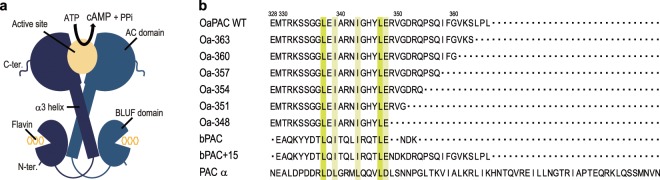
Structure of OaPAC, and amino acid sequences of the C-terminal region of PACs and their mutants. (**a**) Schematic representation of OaPAC. (**b**) Amino acid sequences of the C-terminal region of OaPAC (NCBI, WP_015149803.1), deletion mutants of OaPAC (Oa-363, Oa-360, Oa-357, Oa-354, Oa-351, and Oa-348), bPAC (GenBank: GU461306.2), bPAC mutant (bPAC + 15), and PAC α-chain from Euglena gracilis (PACα). Amino acids highlighted in yellow represent conserved ones.

The structures of OaPAC and bPAC are similar, both forming homo-dimers, and each subunit having the BLUF domain at the N-terminus and AC domain at the C-terminus (^10,15^, Fig. 1a). Two α3-helices of the BLUF domain form a bundle by hydrophobic interaction and reach the AC domain. An active site is located in the interface between AC domains, where cAMP is produced from ATP.

Several studies have been conducted to elucidate the light-activation mechanism of OaPAC and bPAC. Interactions between flavin and neighboring amino acids at the BLUF domain have been reported to vary depending on the irradiated blue light, although structural changes of the BLUF domain during light activation are only subtle according to structural studies^10,15–17^. Studies on the crystal structures of OaPAC and bPAC showed that overall structural changes were also small^10,15,17^. Therefore, it is generally accepted that subtle changes of the BLUF domain, in response to blue light, would induce small allosteric changes of the active site in the AC domain, which produces cAMP from ATP. Moreover, in OaPAC, mutational studies have suggested that the α3-helix bundle at the BLUF domain is related to the transmission of a light-sensing signal from the BLUF domain to the active site^10^. In bPAC, crystal structures have suggested conformational changes in β4 and β5-sheets of the AC domain to regulate the state of the active site^15^.

While the light-activation mechanism has been clarified to some extent, the regulatory mechanism of adenylyl cyclase activity in PACs has not yet been clearly understood. Why the activity is about 100 times higher in bPAC than in OaPAC, despite their similarity in amino acid sequence and structure, remains to be answered. Some studies have suggested the photocycle of the BLUF domain to affect the overall activity^7,16^; however, whether only that domain can influence the regulatory activity remains unresolved.

In this study, we aimed to investigate the mechanism of regulating adenylyl cyclase activity in OaPAC, focusing on the C-terminal region. The sequence of this region is the only notable difference between OaPAC and bPAC, although no functional or structural information regarding the C-terminal region of PAC is available to date. We prepared several C-terminal deletion mutants of OaPAC and investigated their properties. These mutants showed different photoactivities depending on their C-terminal lengths, suggesting that the C-terminal region correlates to OaPAC activity. In addition, as these mutants are activated by different light intensities and produce varying cAMP yields at the same illumination intensity, they could be used in a wide range of experiments as optogenetic tools.

## Results

### Amino acid sequence of PACs

Figure 1b shows the amino acid sequence of the C-terminal region of PACs and their mutants. A conserved residue closest to the C-terminus in OaPAC, bPAC, and PAC α-chains from *Euglena gracilis* (PACα) is a glutamic acid (E348 at OaPAC). OaPAC has 18 additional amino acids at the C-terminus, whereas bPAC has only three. To investigate the effect of the C-terminal region on the activity of OaPAC, we prepared six mutants, namely, Oa-363, Oa-360, Oa-357, Oa-354, Oa-351, and Oa-348, in which 3, 6, 9, 12, 15, and 18 amino acids of the C-terminal region were deleted, respectively.

### The C-terminal region inhibits the adenylyl cyclase activity of OaPAC

We examined the photoactivated adenylyl cyclase activities of the C-terminal-deleted OaPAC mutants in HEK293 cells using GloSensor-22F cAMP, a luciferase-based cAMP reporter. HEK cells co-expressing OaPAC and GloSensor-22F cAMP were stimulated with blue light, and the resultant transient increase in cAMP was monitored by detecting luminescence from GloSensor-22F cAMP every 1 minute. In addition, as OaPAC and red fluorescent protein (RFP) were co-expressed using a single vector containing 2 A self-cleaving peptides, which are known to produce equal amounts of multiple proteins^18,19^, we considered RFP fluorescence as OaPAC expression level.

**Figure 2:**
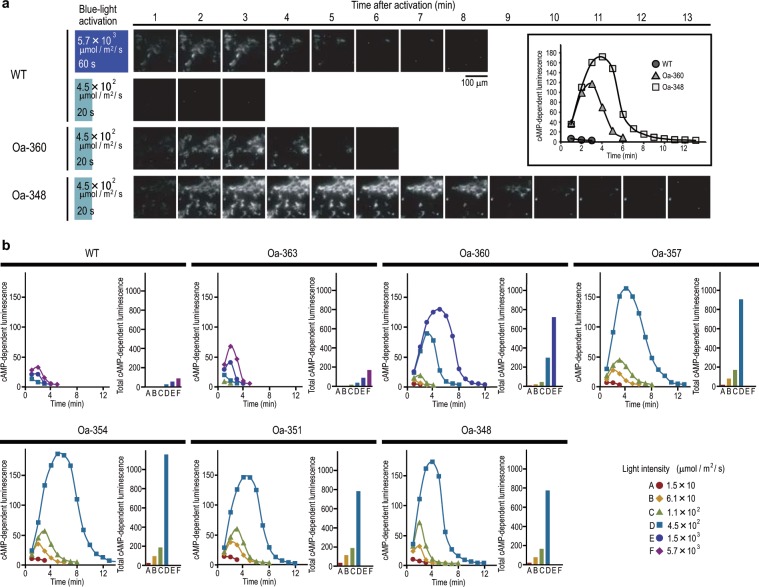
C-terminal deletion mutants showed high photoactivity. HEK cells expressing WT, Oa-360, and Oa-348 were exposed to blue light, and the resulting cAMP-dependent luminescence was detected. (**a**) Representative sequential luminescence images after illumination. The inset shows quantitative plots of luminescence intensities after exposure to blue light at 4.5 × 10^2^ µmol m^−2^ s^−1^ for 20 s. Under the same irradiation condition (4.5 × 10^2^ µmol m^−2^ s^−1^ for 20 s), the luminescence of Oa-348 and Oa-360 was obviously high, while that of the WT was mostly undetectable. The luminescence of the WT was clearly detected by longer exposure to more intense blue light (5.7 × 10^3^ µmol m^−2^ s^−1^, 60 s). (**b**) Time course of cAMP-dependent luminescence intensities (left) and integrated luminescence intensities (right) at indicated blue light intensities. C-terminal deletion mutants were activated by weaker blue light intensities, and they produced a larger amount of cAMP than the WT.

When cells expressing Oa-360 or Oa-348 were illuminated with blue light at 4.5 × 10^2^ µmol m^−2^ s^−1^ for 20 s, a transient increase in luminescence was detected, whereas luminescence from WT was hardly detectable at the same intensity of illumination (Fig. 2a). Higher intensity (5.7 × 10^3^ µmol m^−2^ s^−1^) and longer exposure of blue light (60 s) were required to observe the transient increase in luminescence in WT. In addition, the luminescence from Oa-348 was higher and was observed for a longer time compared to that from Oa-360.

Figure 2b shows adenylyl cyclase activities triggered by various intensities of blue light. The cAMP yield produced in every OaPAC varied with blue light intensity in a dose-dependent manner. Moreover, in the order of Oa-348 > Oa-360 > WT, the intensity of blue light required for photoactivation decreased and the amount of cAMP produced for the same intensity of illumination increased. These results together indicated that photoactivity of OaPAC depends on the number of amino acids at the C-terminus.

**Figure 3:**
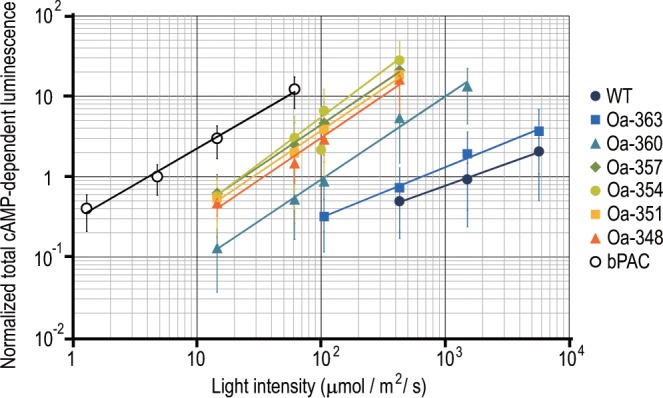
The nine amino acids at the C-terminal critically affect the photoactivity. Dose-response curves for the photoactivities of PACs. cAMP-dependent luminescence of each cell was measured, and the total cAMP-dependent luminescence of each cell was normalized to their expression. Data were collected from different cells, and normalized luminescence was plotted against irradiated intensities. PACs with shorter C-terminal regions produced larger amounts of cAMP. However, the deletion of more than nine amino acids did not further increase their activities. Bars indicate mean ± S.D. (n ≥ 25).

We investigated the photoactivities of all OaPAC mutants at various irradiation intensities (Figs. 2b, 3, S1, S2). Figure 3 shows the normalized dose-response curves for the photoactivities of OaPAC mutants; photoactivities obviously increased as the C-terminal region was shortened. For example, at the blue light intensities of 4.5 × 10^2^ µmol m^−2^ s^−1^, total cAMP-dependent luminescence from Oa-363, Oa-360, and the mutants in which >9 C-terminal amino acids were deleted (Oa-357, Oa-354, Oa-351, and Oa-348) were 1.5, 14, and 30–60 times higher than that from WT, respectively. There was not much difference in photoactivity among the >9 residue-deleted mutants, suggesting that the deletion of >9 residues does not add to the activity significantly. This implies that nine amino acids at the C-terminus of OaPAC have the ability to inhibit its adenylyl cyclase activity.

### The C-terminal region of OaPAC cannot inhibit the activity of bPAC

To investigate the effects of the C-terminal region of OaPAC on the photoactivity of bPAC, which natively has a shorter C-terminus along with higher activity, we prepared a mutant, bPAC + 15, in which 15 amino acids of OaPAC C-terminus were fused to the C-terminus of bPAC (Fig. 1b), and measured the adenylyl cyclase activity of bPAC WT and bPAC + 15 in HEK cells.

**Figure 4:**
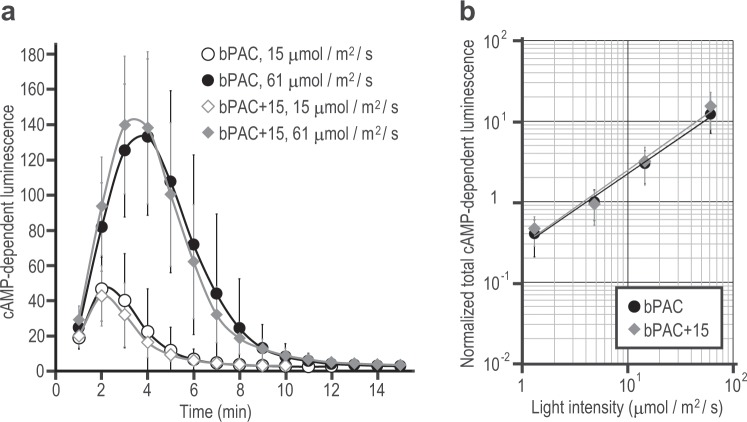
The C-terminal region of OaPAC did not affect the activity of bPAC. HEK cells expressing bPAC or bPAC + 15 were illuminated with blue light, and the cAMP yield was detected as luminescence. (**a**) Representative cAMP-dependent luminescence every 1 minute after blue light illumination is shown at indicated intensities. (**b**) Normalized total cAMP-dependent luminescence of bPAC and bPAC + 15 versus blue light intensities is shown. Data were collected from different cells. Bars indicate mean ± S.D. (n ≥ 21).

The cAMP-dependent luminescence from bPAC + 15 was almost the same as that from bPAC WT (Fig. 4), indicating that the photoactivity of bPAC was not affected by the addition of C-terminal residues from OaPAC. This suggests that inhibition of photoactivity in OaPAC is induced not only by the C-terminal region itself but also by its interaction with some specific site in OaPAC.

### Reduced photoactivity by the C-terminal region occurs independent of the structural changes in the BLUF domain

**Figure 5:**
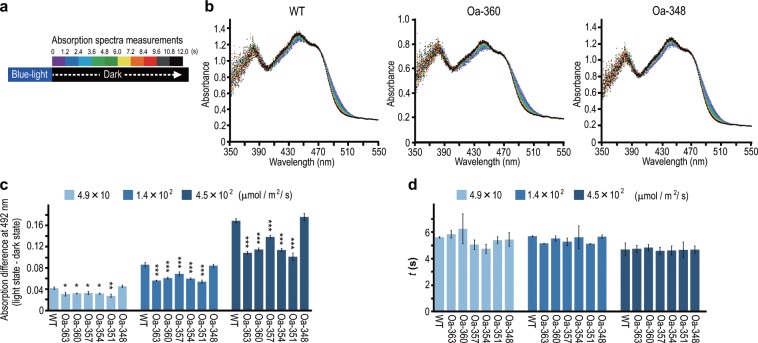
The C-terminal region did not influence the structural changes in the BLUF domain. Structural changes in the BLUF domain, depending on blue-light irradiation, were detected by recording the absorption spectra. (**a**) OaPAC WT and mutants were irradiated with blue light for 20 s, and immediately after, absorption spectra were recorded every 0.4 s. (**b**) Representative absorption spectra of the WT, Oa-360, and Oa-348 at irradiation intensity of 4.5 × 10^2^ µmol m^−2^ s^−1^. Spectra after every 1.2 s are shown in the indicated color in (**a**). (**c**) Absorption differences between light-adapted states and dark-adapted states at 492 nm for each irradiated intensity. Bars indicate mean ± S.D. (n ≥ 3). Absorption differences were analyzed using one-way ANOVA (4.9 × 10 µmol m^−2^ s^−1^; *F* (6, 14) =  14.008, *P* < 0.0001. 1.4 × 10^2^ µmol m^−2^ s^−1^; *F* (6, 17) = 42.151, *P* < 0.0001. 4.5 × 10^2^ µmol m^−2^ s^−1^; *F* (6, 43) =  279.281, *P* < 0.0001) followed by the Tukey–Kramer test (*P < 0.05; ** *P* < 0.01; ***P < 0.0001 vs. WT). (**d**) Time constants of structural changes from light-adapted states to the dark-adapted states at 492 nm for each irradiated intensity. Bars indicate mean ± S.D. (n ≥ 3). The time constants of rates were analyzed using one-way ANOVA, and no statistically significant difference was found between the WT and mutants (4.9 × 10 µmol m^−2^ s^−1^; *F* (6, 14) =  1.776, *P* =  0.176. 1.4 × 10^2^ μmol m^−2^ s^−1^; *F* (6, 17) =  1.103, *P*  =  0.401. 4.5 × 10^2^ µmol m^−2^ s^−1^; *F* (6, 43) =  0.294, *P*  =  0.936).

To clarify the mechanism of reduced photoactivity by the C-terminal region of OaPAC, we examined whether the structure of the C-terminal region affects light-dependent structural changes in the BLUF domain. The structure of the BLUF domain in OaPAC is known to change in response to blue light, which leads to a shift of its absorption spectrum; wavelengths of the absorption bands are longer in a light-adapted state than in a dark-adapted state^10^. We measured the absorption spectra of OaPAC WT and mutants every 0.4 s, immediately after blue light was turned off, and found a spectral shift from the light-adapted state to the dark-adapted state (Fig. 5a).

Whole absorption spectra of all mutants, as well as WT, were shifted similarly from longer wavelength to shorter wavelength (Fig. 5b). We calculated the differences in the absorbance at 492 nm, since the difference was maximum at this wavelength (^10^ Fig. 5c). Although some mutants showed statistically significant differences with respect to the WT in absorbance, these differences were not C-terminal dependent differences as seen in the adenylyl cyclase activities. In other words, difference in absorbance was not related to C-terminal length. In addition, time constants of structural changes from light-adapted states to dark-adapted states at 492 nm did not significantly differ (Fig. 5d), indicating that the WT and mutants required mostly identical time periods to change between the states. These results together implied that the C-terminal region does not affect the structural changes in the BLUF domain, thus indicating that the reduction in activity by the C-terminal region is independent of the BLUF domain.

## Discussion

In this paper, we first established the yet unknown role of the C-terminal region of OaPAC in inhibiting its cyclase activity. Since the structure of the C-terminus has not yet been determined, this aspect has not been studied much yet.

Previous studies on OaPAC and bPAC had reported the states of the BLUF domain, α3-helix bundle at the BLUF domain, and the active site of the AC domain to influence the adenylyl cyclase activity^10,15,16^. Here, we discovered for the first time that the C-terminal region also influences its activity. The deletion of the C-terminal region induced higher adenylyl cyclase activity (Figs. 2, 3), suggesting that the C-terminal region inhibits the adenylyl cyclase activity. In Fig. 3, which indicates total cAMP-dependent luminescence versus illuminated blue light intensity, the approximate curve of every deletion mutant was not simply shifted from that of the WT to react to weaker light intensity, but their slopes were also larger than that of the WT. These results indicated that the deletion of the C-terminal region does not only make OaPAC more sensitive to weak light but also increases the enzyme activity. Therefore, the C-terminal region is suggested to inhibit the process of cAMP production.

Mutants in which more than nine amino acids had been deleted from the C-terminal region showed high adenylyl cyclase activity, suggesting the nine amino acids to be important for inhibiting the activity. A similar phenomenon regarding the inhibition of activity by the C-terminal region has also been reported in a soluble adenylyl cyclase (sAC). In sAC, there is an isoform that consists of two catalytic domains (C1 and C2) and lacks almost the entire C-terminal region^20,21^. The adenylyl cyclase activity of the isoform was reported to be 10 times higher than that of the WT, and nine amino acids just behind the C2 domain affected its activity^22^. This property of sAC is similar to that of OaPAC, suggesting the mechanism of activity regulation to be similar in the two proteins.

**Figure 6:**
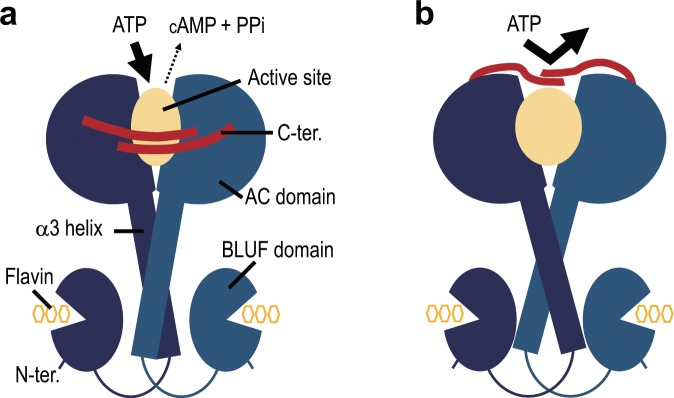
Model of inhibition of adenylyl cyclase activity by the C-terminal region. (**a)** The C-terminal region inhibits the conformational changes of the AC domain, thereby preventing cAMP production from ATP. (**b)** The C-terminal region inhibits ATP binding to the active site.

Considering the properties of sAC, we next discuss how the C-terminal region of OaPAC might influence the adenylyl cyclase activity (Fig. 6). Most of the nine key residues at the C-terminus are hydrophobic in both OaPAC and sAC, although there is no homology between them; seven of the nine amino acids are hydrophobic in OaPAC and six of the nine amino acids are hydrophobic in sAC. Due to the high hydrophobicity of the C-terminal region, there is a possibility that the C-terminal region would enter into the OaPAC structure or interact with another site by hydrophobic interactions. Since the C-terminal region of OaPAC did not affect the BLUF domain (Fig. 5), the relevant interaction site would probably be in the AC domain. The C-terminal region seems to interact with a site that is crucial for conformational changes in the AC domain, which restricts OaPAC from producing cAMP from ATP (Fig. 6a). Alternatively, the C-terminal region may interact with the ATP-binding site in the active site, which is reported to be hydrophobic^15,23^, thereby preventing ATP from binding there (Fig. 6b).

Recently, the structure of a bovine membrane-integral adenylyl cyclase (tmAC) was solved^24^, which revealed that the C-terminal region of tmAC regulated cAMP production by occluding both the active site and the adjacent allosteric site. Sequences of the catalytic domains of tmAC were homologous to those of OaPAC and sAC, although the C-terminal region of tmAC was not homologous to the others and was not even hydrophobic. Similar to that of tmAC, the C-terminus of OaPAC might also influence both conformational changes of the AC domain, as well as ATP-binding, there. Identification of the interaction site would give us clearer insights into the regulatory mechanism of OaPAC by the C-terminal region.

This report shows that the C-terminal region is the most crucial site for inducing differences in activity between OaPAC and bPAC, since the activity of the bPAC-like deletion mutant of OaPAC (Oa-351) was closer to that of WT bPAC (Fig. 3). However, a small difference in activity remained, indicating that another site, other than the C-terminal region, could also be related to the activity in OaPAC. Mutations in the BLUF domain of OaPAC and bPAC have been reported to cause changes in activity^10,16^, implying that the BLUF domain affects the activity. Therefore, this domain may be considered to control light sensitivity as well. Several sites seem to contribute to the regulation of activity, and differences in the activities between OaPAC and bPAC arise from different contributions of these sites.

The difference in photoactivity between OaPAC and bPAC might have arisen from differences in the environment where *Oscillatoria acuminata* and *Beggiatoa* sp. exist. In a microbial mat comprising several layers of specific types of microorganisms, cyanobacteria containing *Oscillatoria* sp. exist near the surface layer^25–27^, whereas sulfide-oxidizing bacteria such as *Beggiatoa* sp. exist in a deeper layer, where light is faint due to its absorption by the upper layers. Therefore, OaPAC in *Oscillatoria acuminata* is exposed to a higher intensity of light than bPAC in *Beggiatoa* sp. Such a difference in light intensity of the environment might induce different photosensitivities in PACs.

Our finding is expected to promote the use of PAC as an optogenetic tool. To date, optogenetic use of PACs has been limited given that intense blue light is required for activating OaPAC, which might damage cells and flavins. In contrast, bPAC is overactivated even by weaker blue light. The OaPAC mutants in this study were activated with weaker blue light, which does not activate OaPAC WT. Therefore, the mutants enable us to regulate cAMP levels with less damage and without requiring excessive light. Moreover, since these mutants showed various cAMP yields for the same blue light intensity, we may even select suitable mutants depending on the purpose of study or cell types. For generating more useful PACs as optogenetic tools, further elucidation of the regulatory mechanism behind photoactivation is required.

## Methods

### Constructs and mutants

PAC genes with Rudolph-RFP and 2A-peptides at the N-terminus (RFP-2A-PACs) were cloned into pEBMulti-Hyg vectors (Fujifilm Wako Pure Chemical Corporation, Osaka, Japan) for expression in HEK293 cells. The GloSensor-22F cAMP gene (Promega Corporation, Fitchburg, WI, USA), a luciferase-based cAMP reporter, was cloned into a pEBMulti-Neo vector (Fujifilm Wako Pure Chemical Corporation, Osaka, Japan) for monitoring the cAMP level in HEK293 cells. PAC genes with a histidine-tag at the N-terminal were cloned into pQE-30 vectors for protein purification from *Escherichia coli*. Each gene was codon-optimized for HEK cells or *E. coli*. C-terminal deletion mutants of OaPAC and mutants with 15 OaPAC C-terminal residues fused to the C-terminus of bPAC were generated using an In-Fusion HD Cloning kit (TaKaRa, Shiga, Japan).

### Protein expression in HEK cells

PACs were expressed in HEK293 cells that stably expressed a GloSensor-22F cAMP, as previously described^10^. Briefly, pEBMulti-Neo vector encoding the GloSensor-22F cAMP gene was transfected into HEK293 cells using FuGENE HD (Promega K.K., Tokyo, Japan), and the cells stably expressing GloSensor-22F cAMP were established with antibiotic selection and single-cell cloning. The pEBMulti-Hyg vectors encoding RFP-2A-PACs genes were transfected into HEK293 cells and RFP fluorescence was detected as an indicator of PAC expression since the 2A peptides ensure equal expression of PAC and RFP^18,19^.

### Monitoring cAMP generation in HEK cells

cAMP produced by PACs in HEK293 cells were monitored as previously described^10^. Briefly, several hours before the experiments, luciferase substrate was added to CO_2_ Independent Medium (Thermo Fisher Scientific K.K. Tokyo, Japan), containing 4 mM glutamine and 10% FBS, and HEK293 cells were added to a final concentration of 0.12 mg/ml in the dark. The cells, set on an inverted microscope (TE300, Nikon Instruments Inc., Tokyo, Japan) equipped with an objective (S Fluor 20x N.A. of 0.75), were exposed to actinic blue light from a LED projector module (Luxeon Rebel Royal Blue 447.5 nm, full width at half maximum of 20 nm [Philips Lumileds Lighting Company, San Jose, CA, USA] equipped with an aspheric condenser lens [Φ of 25 mm, F of 20 mm; ACL2520U, Thorlabs Inc., Newton, NJ, USA]) for 20 s. Immediately after the blue light activation, cellular luminescence due to cAMP generation was captured into sequential images using an EM-CCD camera (ImagEM C9100-13; Hamamatsu Photonics K.K., Hamamatsu, Japan) close to photon-counting conditions (frame exposure of 60 s, EM gain of 250), until cAMP-dependent luminescence was no longer detected. At the same field, fluorescence images from RFP, excited with green-light (by using the Texas-Red fluorescence filter module), were obtained to measure the expression levels of PACs. cAMP-dependent luminescence intensities and fluorescence intensities of RFP in every cell were quantified using ImageJ software. Cells showing excessively bright luminescence, saturated in the EM-CCD camera, and those with continuous and independent abnormal luminescence after blue light illumination, were excluded. cAMP-dependent luminescence intensities were normalized to fluorescence intensities of RFP.

### Protein purification

pQE-30 vectors encoding OaPAC WT or its mutants, carrying an N-terminal histidine tag, were transformed into *E. coli* XL-Blue (Nippon Gene Co., Ltd., Tokyo, Japan) and overexpressed with 0.5 mM isopropyl β-d-1-thiogalactopyranoside for 3 h. The harvested cells were re-suspended in lysis buffer (50 mM NaH_2_PO_4_ [pH 7.0], 300 mM NaCl, and protease inhibitors [cOmplete, EDTA-free Protease Inhibitor Cocktail; Roche, Basel, Switzerland]) and disrupted using lysozyme (final concentration of 1 mg/ml) and sonication. The crude sample was centrifuged at 4,000 × *g* for 10 min at 4 °C. Flavin mononucleotide was added to the supernatant (final concentration of 10 mM) and incubated overnight at 4 °C in the dark. Co^2+^ affinity gel beads (TALON Metal Affinity Resins; TaKaRa Bio USA, Inc., CA, USA), equilibrated with normal buffer (50 mM NaH_2_PO_4_ [pH 7.0], 300 mM NaCl), were added to the sample and incubated for 2 h at 4 °C in the dark to bind them to the histidine tag. The beads were then washed off with wash buffer (50 mM NaH_2_PO_4_ [pH 7.0], 300 mM NaCl, 5 mM imidazole), and the OaPAC proteins were eluted with elution buffer (50 mM NaH_2_PO_4_ [pH 7.0], 300 mM NaCl, 150 mM imidazole). Fractions containing the proteins were loaded onto a PD-10 column (GE Healthcare UK Ltd., Buckinghamshire, England) and eluted with normal buffer.

### Spectroscopy

A rapid-scan spectrophotometer was constructed by arranging an actinic blue light projection LED module (Luxenon Rebel Royal Blue 447.5 nm, full width at half maximum of 20 nm [Philips Lumileds Lighting Company] equipped with an aspheric condenser lens, Φ of 25 mm, F of 20 mm [ACL2520U; Thorlabs Inc.]), a tungsten projection lamp for absorption spectroscopy (262154; Olympus Corp., Tokyo, Japan), a Photonic Multichannel Analyzer (C7473-36; Hamamatsu Photonics K.K.), and an electro-magnetic shutter module (C-79; Chuo Precision Ind. Co. Ltd., Tokyo, Japan) in the optical path. This rapid-scan spectrophotometer was driven by a multiple array of electric relays (R4 ichan; Data Six Co., Ltd., Kobe, Japan). OaPAC solution in a quartz cuvette (diameter of 1 cm) was placed in the optical path and irradiated by actinic blue light (447.5 nm) for 20 s. Immediately after this, the absorption spectrum between 350 nm and 550 nm was recorded using the rapid-scan spectrophotometer, at every 0.4 s, until OaPAC was fully relaxed. Absorption differences and time constants of conversion from light-adapted states to dark-adapted states at 492 nm were analyzed using one-way ANOVA, followed by the Tukey–Kramer test.

## Supplementary information

Supplementary Information.
