# Supplementary material for: The C-terminal region affects the activity of photoactivated adenylyl cyclase from Oscillatoria acuminata
Source: Sci Rep. 2019 Dec 30;9:20262. doi: 10.1038/s41598-019-56721-3 (PMC6937261; doi:10.1038/s41598-019-56721-3)
Supplement: Supplementary file 1 — Supplementary Information. [file 41598_2019_56721_MOESM1_ESM.pdf]

Supplementary Information

The C-terminal region affects the activity of photoactivated adenylyl cyclase from *Oscillatoria acuminata*

Minako Hirano[1]*, Masumi Takebe[2], Tomoya Ishido[3], Toru Ide[3], Shigeru Matsunaga[2]*

[1] Bio Photonics Laboratory, The Graduate School for the Creation of New Photonics Industries, 1955-1 Kurematsu Nishi-ku, Hamamatsu, Shizuoka 431-1202, Japan

[2] Central Research Laboratory, Hamamatsu Photonics K.K., 5000 Hiraguchi Hamakita-ku, Hamamatsu, Shizuoka 434-8601, Japan

[3] Graduate School of Interdisciplinary Science and Engineering in Health Systems, Okayama University, 3-1-1 Tsushima-naka, Kita-ku, Okayama-shi, Okayama 700-8530, Japan

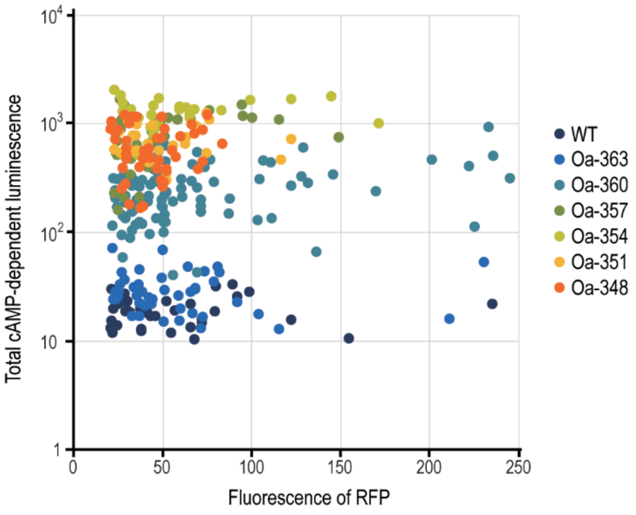

Figure S1. Total cAMP-dependent luminescence within each cell against fluorescence intensity of RFP (indicating OaPAC expression level). HEK cells expressing OaPAC or their mutants were illuminated with blue light at 4.5×10^2 µmol m^{-2} s^{-1} for 20 s. ($n \geq 27$).

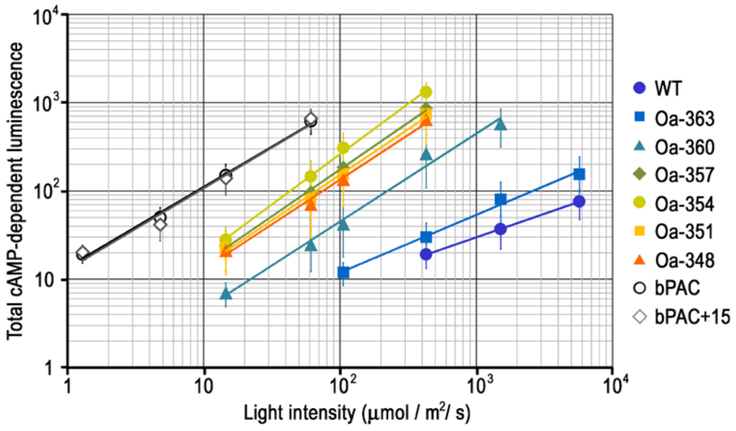

Figure S2. Total cAMP-dependent luminescence against blue light intensities. HEK cells expressing PACs were illuminated with blue light at various intensities and the produced cAMP was detected as luminescence. Bars indicate mean ± s.d. ($n \geq 25$).
